# HTLV-1 world distribution and estimation of the number of asymptomatic infected carriers

**DOI:** 10.1186/1742-4690-11-S1-O14

**Published:** 2014-01-07

**Authors:** Antoine Gessain, Olivier Cassar

**Affiliations:** 1 Institut Pasteur, Unité d’Epidémiologie et Physiopathologie des Virus Oncogènes, Département de Virologie, Paris, France; 2 CNRS, UMR 3569, Paris, France

## 

HTLV-1, identified 32-years-ago, is present throughout the world with clusters of high endemicity such as Southwestern Japan, sub-Saharan Africa, South America, the Caribbean area and foci in Middle-East and Australo-Melanesia. The origin of this puzzling geographic/ethnic distribution is probably linked to founder effects in some groups with the persistence of a high viral transmission rate. Twenty-years-ago, de-Thé and Bomford estimated the number of HTLV-1 carriers to be 10-20 millions. At that time, large regions had not been investigated, few population-based studies were available and the assays used for HTLV-1 serology were not specific enough. Despite the lack of data for some large areas of the world, and the fact that most of the HTLV-1 studies concern blood donors, pregnant women or high-risk groups, we tried to revisit HTLV-1 world distribution and estimate, for the first time, the number of HTLV-1 infected persons by continent, regions and countries when possible (*Gessain and Cassar*, *Front Microbiol*, *2012*). Our estimation was based (i) on most reliable available publications regarding the HTLV-1 prevalence and (ii) the global repartition of individuals by age and sex in each studied country. Our best estimates range from 5-10 million HTLV-1 infected individuals. However, these results were based solely on the 1.5 billion individuals living in the known endemic areas or regions with reliable epidemiological data. Correct estimates in other highly populated regions (China, India, North and East Africa,…) is currently not possible, thus, the current number of HTLV-1 carriers is very probably much higher.

